# Supplementary material for: Luck and Intentional Action: A Causal Account
Source: Open Mind (Camb). 2026 Jun 17;10:857–83. doi: 10.1162/OPMI.a.361 (PMC13327788; doi:10.1162/OPMI.a.361)
Supplement: Supplementary file 1 [file opmi-10-857-s001.pdf]

Supplementary Information for: Luck and intentional action: a causal account

Supplementary Information for: Luck and intentional action: a causal account

Supplementary analysis for Experiment 1.

Here we analyse results from Experiment 1 in a between-subjects analysis by looking at only at the first trial for each participant. Figure S1 displays the results.

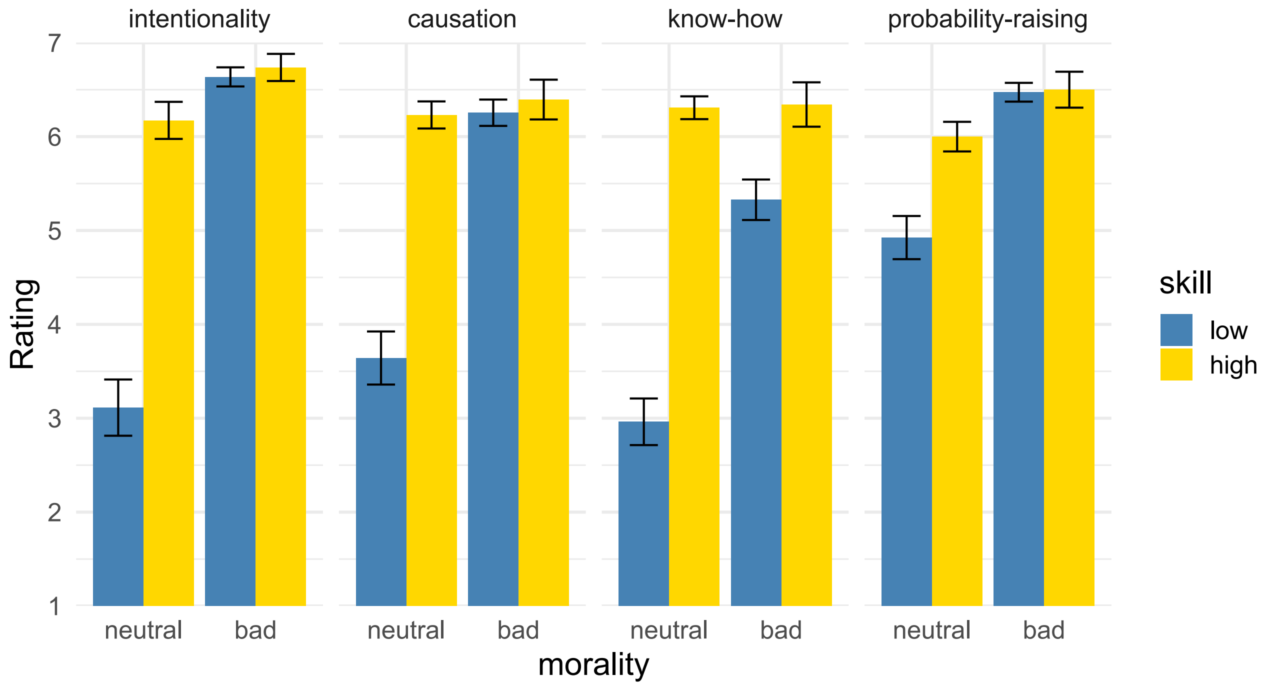

Figure S1

Mean judgments of intentionality, causation, know-how and probability-raising as a function of skill and morality, Study 1, restricted to the first trial for each participant. Error bars display standard errors.

A 2*2 Anova for intentionality judgments showed a main effect of skill, $F(1,194) = 66$, $p < .001$, a main effect of morality, $F(1,194) = 98$, $p < .001$, as well as an interaction, $F(1,194) = 50$, $p < .001$.

For causal judgments, there was a main effect of skill, $F(1,194) = 51$, $p < .001$, a main effect of morality, $F(1,194) = 46$, $p < .001$, and an interaction, $F(1,194) = 35$, $p < .001$. For know-how judgments, there was a main effect of skill, $F(1,194) = 114$, $p < .001$, a

main effect of morality, $F(1,194) = 28$, $p < .001$, and an interaction, $F(1,194) = 30$, $p < .001$. For probability-raising judgments, there was a main effect of skill, $F(1,194) = 11$, $p < .001$, a main effect of morality, $F(1,194) = 34$, $p < .001$, and an interaction $F(1,194) = 9$, $p = .003$.

We also ran a multiple regression predicting participants' judgments of intentionality from their judgments of causation, know-how and probability-raising. We find that causation is the best predictor of intentionality judgments, $\beta = .51$, $p < .001$, followed by know-how, $\beta = .28$, $p < .001$. There was a positive but non-significant effect of probability-raising, $\beta = .09$, $p = .08$.

Questions for morally bad scenario in Study 2

In the Means condition, the questions were:

-intentionality: 'Joan intentionally made the arrow shoot down path eight'.

-causation: 'The arrow shot down path eight because Joan wanted to shoot the arrow down path eight'.

-know-how: 'Joan knows how to make the arrow shoot down path eight'.

-probability-raising: 'Pulling the lever increased the probability of the arrow shooting down path eight'.

In the Ends condition:

-intentionality: 'Joan intentionally killed Bill'.

-causation: 'Bill died because Joan wanted to kill him'.

-know-how: 'Joan knows how to kill Bill'.

-probability-raising: 'Pulling the lever increased the probability of Bill dying'.

Supplementary Experiments

Experiments S2a-b

Studies S2a and S2b investigate the Kraemer effect (as in Study 2) separately for the morally neutral (Study S2a) and the morally bad scenario (Study S2b). The study reported in the main text was meant to replicate the findings from these studies with a

slightly larger sample.

Methods

We used two different vignettes, adapted or reproduced verbatim from Pavese and Henne (2023). We performed a separate pre-registered study for each vignette, and report the results of the two studies jointly for simplicity.[1] One vignette used a morally neutral action (Study S2a) and the other vignette used a morally bad action (Study S2b). Specifically, Study S2a used an adapted version of the 'game show' vignette from Pavese & Henne's Experiment 1, and Study S2b used their 'moral' vignette, reproduced verbatim from their Experiment 5.

Methods and stimuli were identical to Study 2 (see main text), with the following exceptions. Participants answered the intentionality question first, followed by the causation, know-how and probability-raising questions, presented in random order on the next page (this is the reverse of Study 2 in the main text). Comprehension questions for Study S2a were 'Which color is most likely to be selected?' and 'Can one predict the outcome with 100% certainty?'.

Median participation time was 4 minutes and participants were compensated £0.50 for participation. The studies were pre-registered at https://osf.io/xvf9g/?view_only=17e8e050bb864b3a9815e159f1366ed3 (Study S2a) and https://osf.io/xyz32/?view_only=6b83dfbccf004322933bc57ee9ce293e (Study S2b).

Participants

Pavese and Henne (2023) obtained robust evidence for a Kraemer effect with about 200 participants (see their Experiment 3). Their experiment was within-subject. Since we ask more questions, we decided to adopt a between-subjects design. To account for the between-subjects design, we decided to increase our sample size to the extent allowed by financial resource constraints, and decided to target 300 participants per vignette.

[1] Because each pre-registration was specific to a particular study, analyses that refer to the whole dataset should technically be considered exploratory.

For Study S2a, we recruited 298 US residents from Prolific (146 female, 2 other, mean age: 38, SD: 13). Following our pre-registered exclusion criteria, we analyzed data for 164 participants who successfully completed two comprehension questions.[2]

For Study S2b, we recruited 295 US residents (148 female, 1 other; mean age: 39, SD: 15) from Prolific. We excluded from analysis participants who responded to a white-ink question (N=2) or who failed a comprehension question (N=19), for a final sample of 274 participants.[3]

Results

Results are displayed on Figure S2. We analyzed intentionality judgments with a 2*2 Anova. Replicating the results of Pavese and Henne (2023), there was a main effect of condition, $F(1,434) = 67$, $p < .001$, with participants assigning higher intentionality to the ends (M=4.77, SD=2.31) relative to the means (M=3.24, SD=2.27). There was also a main effect of morality, $F(1,434) = 147$, $p < .001$, with participants assigning higher intentionality in the morally bad (M=4.92, SD=2.26) relative to the morally neutral vignette (M=2.55, SD=1.89). There was also an interaction, $F(1,434) = 14$, $p < .001$: the effect of condition was larger in the morally bad relative to the morally neutral vignette.

Crucially, we find a similar pattern for judgments of causation. There was a main

[2] This is an unusually high exclusion rate. Looking at the comprehension items for that study, we find that one question ('Can one predict the outcome with 100% certainty?') was correctly answered by only 182 out of 298 participants. The other question ('Which color is most likely to be selected') had a much better success rate, being correctly answered by 270 participants. We speculate that the first question was poorly worded, especially for a non-scientific audience. In an exploratory analysis on the full sample, we find mostly identical results, with the exception that the effect of condition on causation ratings is only marginally significant in the morally neutral condition, $p = .08$. Study S2b used clearer comprehension questions.

[3] Study S2b featured two comprehension questions presented on a separate page after the causation, know-how and probability-raising questions. Participants were asked whether Joan felt sad that Bill had died (True / **False**) and on which path Bill was standing (Three/**Eight**/Ten).

effect of condition, $F(1,434) = 73$, with participants assigning higher causation in the ends (M=4.45, SD=2.29) relative to the means condition (M=2.89, SD=2.12). There was also a main effect of morality, $F(1,434) = 131$, $p < .001$, with participants assigning higher causation in the morally bad (M=4.51, SD=2.27) relative to the morally neutral vignette (M=2.34, SD=1.75). There was also an interaction, $F(1,434) = 17$, $p < .001$: the effect of condition was larger in the morally bad vignette.

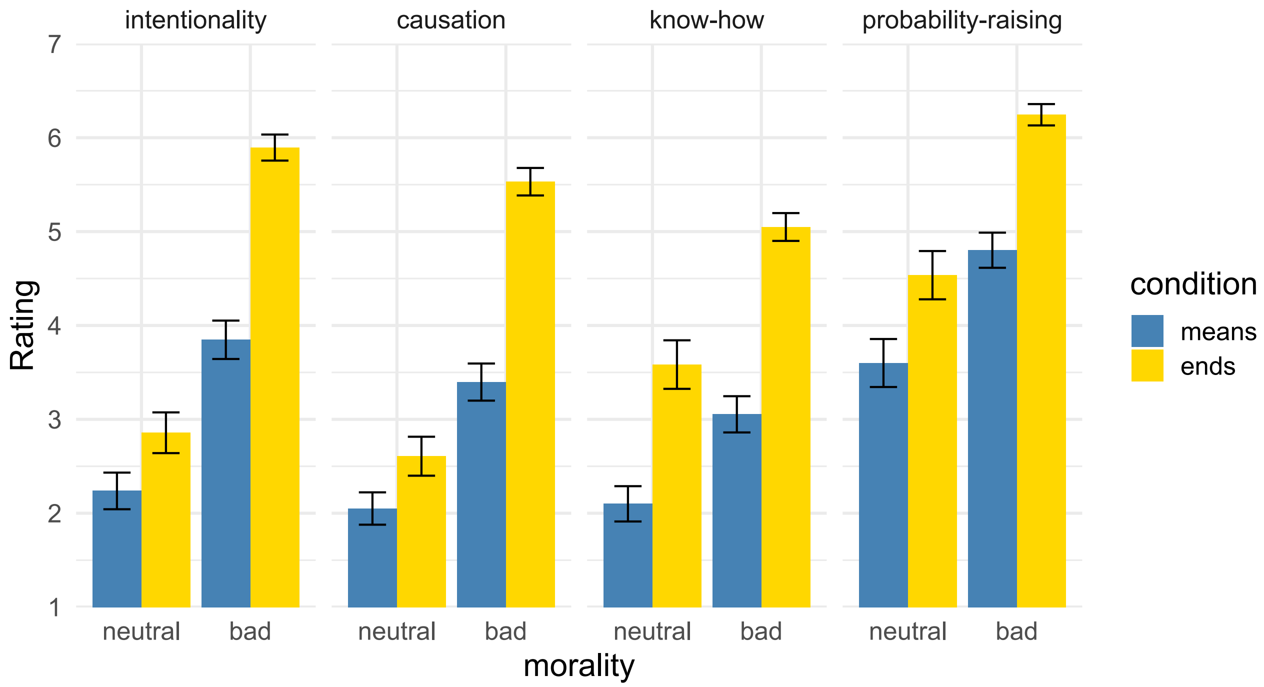

Figure S2

Mean ratings for each question, as a function of morality and condition, Studies S2a-b. Error bars represent standard errors.

For know-how judgments, we replicated Pavese & Henne's finding of a main effect of condition, $F(1,434) = 37$, $p < .001$, with participants assigning higher know-how for the ends (M=4.51, SD=2.13) relative to the means (M=2.69, SD=2.07). There was also a main effect of morality, $F(1,434) = 88$, $p < .001$, with participants assigning more know-how to the agent in the morally bad (M=4.09, SD=2.23) relative to the morally neutral vignette

(M=2.86, SD=2.19). There was no interaction between condition and morality, however, F(1,434) = 1.6, p = .20.

Finally, for probability-raising judgments, we also find a main effect of condition, F(1,434) = 44, p < .001, and a main effect of morality, F(1,434) = 55, p < .001, but no interaction, F(1,434) = 1.7, p = .20.

To assess whether the Kraemer effect is found within each vignette, we also ran pre-registered independent-samples t-tests within each study. In the morally neutral vignette, intentionality judgments were higher for the ends (M=2.86, SD=1.99) than for the means (M=2.24, SD=1.74), t(160.9) = 2.12, p = .035. The same pattern held for judgments of causation (ends: M=2.61, SD=1.90, means: M=2.05, SD=1.54, t(158.03) = 2.07, p = .04), know-how (ends: M=3.58, SD=2.37, means: M=2.10, SD=1.68, t(149.9) = 4.64, p < .001) and probability-raising (ends: M=4.54, SD=2.36, means: M=3.60, SD=2.29, t(161.9) = 2.58, p = .01).

In the morally bad vignette, intentionality judgments were higher for the ends (M=5.90, SD=1.66) than the means (M=3.85, SD=2.34), t(232.2) = 8.28, p < .001. The same pattern held for judgments of causation (ends: M=5.53, SD=1.75, means: M=3.40, SD=2.26, t(244.5) = 8.68, p < .001), know-how (ends: M=5.05, SD=1.77, means: M=3.05, SD=2.21, t(249.3) = 8.20, p < .001) and probability-raising (ends: M=6.24, SD=1.36, means: M=4.80, SD=2.14, t(216.6) = 6.59, p < .001).

We also ran a multiple regression predicting participants' intentionality judgments as a function of their judgments of causation, know-how and probability-raising. We find that causation is the best predictor of participants' intentionality judgments, $\beta = .59$, p < .001, followed by know-how, $\beta = .16$, p < .001, and probability-raising, $\beta = .12$, p < .001. We used a participant-level bootstrapping procedure (1,000 resamples) to estimate confidence intervals for the differences between standardized regression coefficients. The analysis suggests a reliable difference between the effect of causation and know-how (95% CI: [.25, .61]), as well as between the effect of causation and probability-raising (95% CI:

[.32, .61]), but there was no reliable difference between the effect of know-how and probability-raising (95% CI: [-.09, .16]).

Supplementary Experiment S2c

In Study S2a we find tentative evidence for a Kramer effect for causal judgment in a morally neutral scenario. Study S2c was designed to replicate that finding. We used a within-subjects design in an attempt to increase statistical power.

Procedure

Participants read the same vignette as in Study S2a. They rated their agreement, on a 1-7 Likert scale (1: strongly disagree; 7: strongly agree) with the following statements:

-'Sally got a green ball because she wanted to get a green ball'.

-'Sally won the car because she wanted to win the car.'

The two statements were presented on the same page. Their order of presentation on the screen was randomized.

After this main phase, participants answered two comprehension questions, presented to participants on the page right after the page containing the main questions. This page featured a reminder of the scenario. The comprehension questions were:

-If Sally had gotten a purple ball, what would have happened? [She would still have won the car / She would have won a different prize / **She would have won nothing**]

-Was Sally disappointed when she got a green ball? [Yes / **No**]

Participant failing either question were excluded from analysis. The design of the study and exclusion criteria were pre-registered at https://osf.io/r4kh2/?view_only=9790223d8f524846aa254b4d1037948f.

Participants

We recruited 199 US residents from Prolific (mean age: 39, SD=13; 101 female). We excluded from analysis 12 participants failing at least one comprehension question, for a final sample of 187 participants.

Results

We find only weak potential evidence for a Kraemer effect for causal judgments in this study, see Figure S3. Causal judgments for the Ends (M=3.20, SD=2.11) were slightly higher than for the Means (M=3.04, SD=2.07), but this effect was only marginally significant, t(186)=1.81, p = .07, two-tailed dependent-samples t-test.

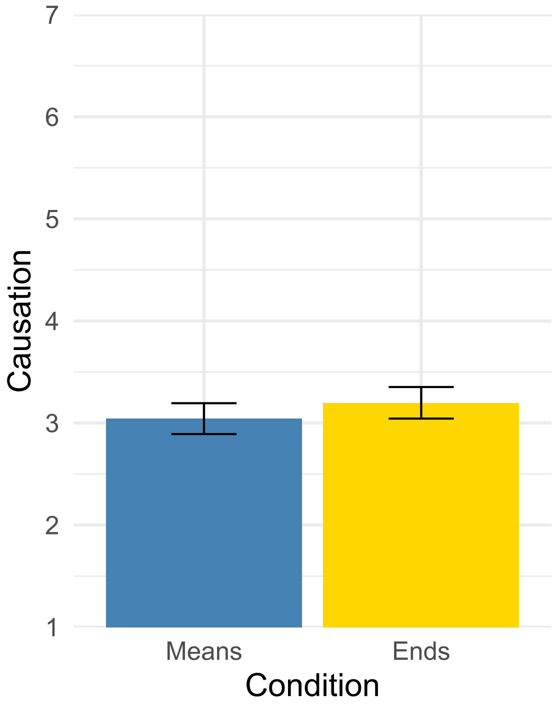

Figure S3

Mean causal judgments as a function of condition, Study S2c. Error bars display standard errors.

Discussion

Overall, although we find strong evidence for a Kraemer effect for causal judgments in a morally bad scenario (Study S2b), the results of Studies S2a and S2c, taken together, only provide very moderate evidence for a corresponding effect in a morally neutral scenario. We again fail to find evidence for the effect in the main text (Study 2); see also Varghese and Henne (2025). In sum, at least for the particular scenario we used, a

Kraemer effect for causal judgment in a morally neutral context is either weak and difficult to detect, or does not exist. We note that while we do find a Kraemer effect for *intentional action* in this scenario, it is quite small.

Supplementary analysis for Study 3

Here was analyze results from Study 3 with between-subjects tests, focusing on the data from the first trial of each participant. Results are displayed in Figure S4.

For intentionality, there was a main effect of Knowledge, $F(1,138) = 14.4$, $p < .001$, a main effect of Process, $F(1,138) = 26.6$, $p < .001$, and an interaction, $F(1,138) = 9.4$, $p = .003$. For causation, there was a main effect of Knowledge, $F(1,138) = 7.2$, $p = .008$, a main effect of Process, $F(1,138) = 12.3$, $p < .001$, and an interaction, $F(1,138) = 7.5$, $p = .007$.

For know-how, there was a main effect of Knowledge, $F(1,138) = 127.9$, $p < .001$, but no effect of Process, $F(1,138) = 1.7$, $p = .19$, and no interaction, $F(1,138) = 0.6$, $p = .45$. For probability-raising, there was no main effect of Knowledge, $F(1,138) = 0.8$, $p = .37$, a main effect of Process, $F(1,138) = 15.2$, $p < .001$, and no interaction, $F(1,138) = 0.002$, $p = .97$.

We ran a multiple regression predicting participants' intentionality judgments on the basis of their judgments of causation, know-how and probability-raising. Causation was the strongest predictor, $\beta = .53$, $p < .001$, followed by Know-how, $\beta = .21$, $p = .003$. There was a positive but non-significant effect of Probability-raising, $\beta = .04$, $p = .54$.

Mediation analyses

Here we report the results of exploratory mediation analyses meant to assess whether causal judgments mediate the effect of our manipulation of luck (this manipulation corresponds to the Skill, Condition (Ends vs Means) and Process manipulations in Studies 1, 2 and 3 respectively). We note that these results should be interpreted with appropriate caution, given the demanding assumptions underlying causal mediation analysis (Rohrer et al., 2022). All mediation models were fit using the 'mediation' package in R (Tingley et al., 2014).

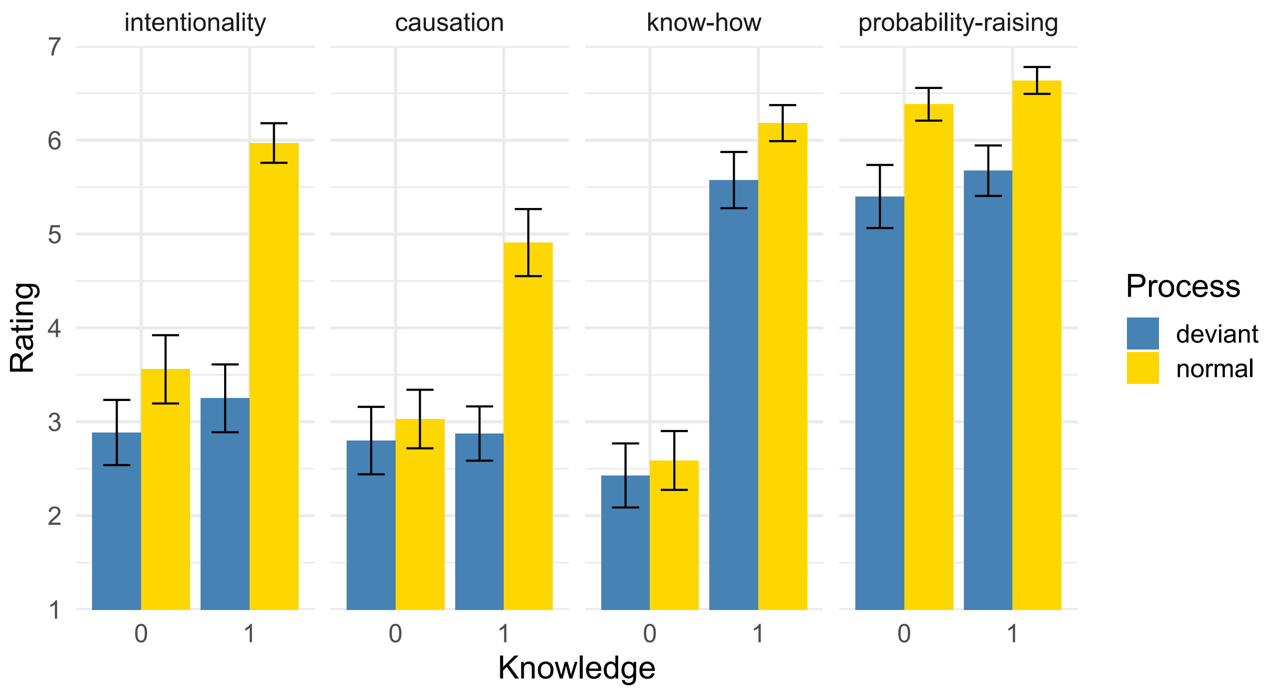

Figure S4

Mean judgments of intentionality, causation, know-how and probability-raising in Study 3, restricted to the first trial seen by each participant. Error bars display standard errors.

Study 1

We ran a mediation analysis to test whether causation mediates the relationship between skill and intentionality; see Figure S5. We fitted mixed-effects models with random intercepts for participants.

There was a significant indirect effect of skill through causation (ACME = 0.93, 95% CI [0.78, 1.10], $p < .001$). The direct effect of skill on intentionality remained significant after controlling for causation (ADE = 0.52, 95% CI [0.36, 0.69], $p < .001$), suggesting partial mediation. The total effect was 1.45 (95% CI [1.24, 1.66], $p < .001$). Causation accounted for approximately 64% of the total effect (95% CI [0.56, 0.73]).

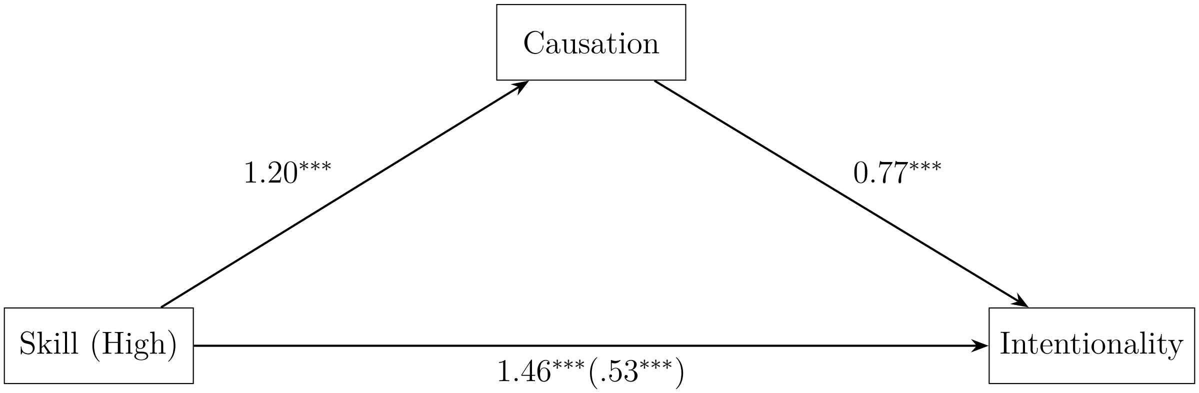

Figure S5

Mediation model for Study 1. Regression coefficients are computed from linear mixed models with participant-level random intercepts. The coefficient in parentheses represents the direct effect of Skill on Intentionality.

Study 2

We ran a mediation analysis to test whether causation mediates the relationship between condition (Ends vs Means) and intentionality; see Figure S6.

There was a significant indirect effect of condition (Ends vs Means) through causation (ACME = 0.85, 95% CI [1.08, 0.61], $p < .001$). The direct effect of condition on intentionality remained significant after controlling for causation (ADE = 1.02, 95% CI [0.78, 1.27], $p < .001$), suggesting partial mediation. The total effect was 1.86 (95% CI [1.53, 2.17], $p < .001$). Causation accounted for approximately 45% of the total effect (95% CI [0.36, 0.55]).

Study 3

We ran a mediation analysis to test whether causation mediates the relationship between process and intentionality; see Figure S7. We fitted mixed-effects models with random intercepts for participants.

There was a significant indirect effect of process through causation (ACME = 0.74, 95% CI [0.52, 0.96], $p < .001$). The direct effect of process on intentionality remained significant after controlling for causation (ADE = 0.77, 95% CI [0.51, 1.02], $p < .001$),

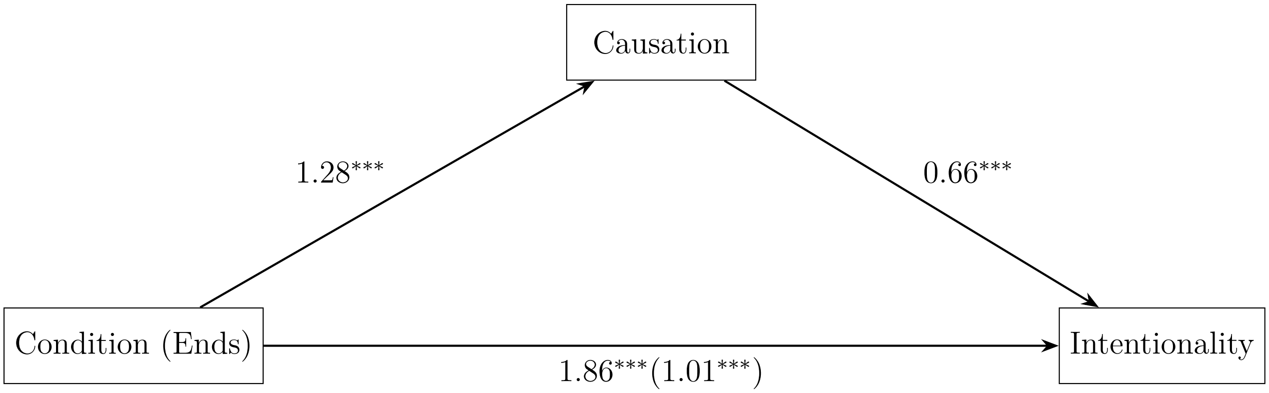

Figure S6

Mediation model for Study 2. The regression coefficient in parentheses represents the direct effect of Condition on Intentionality.

suggesting partial mediation. The total effect was 1.51 (95% CI [1.19, 1.83], p < .001). Causation accounted for approximately 49% of the total effect (95% CI [0.38, 0.61]).

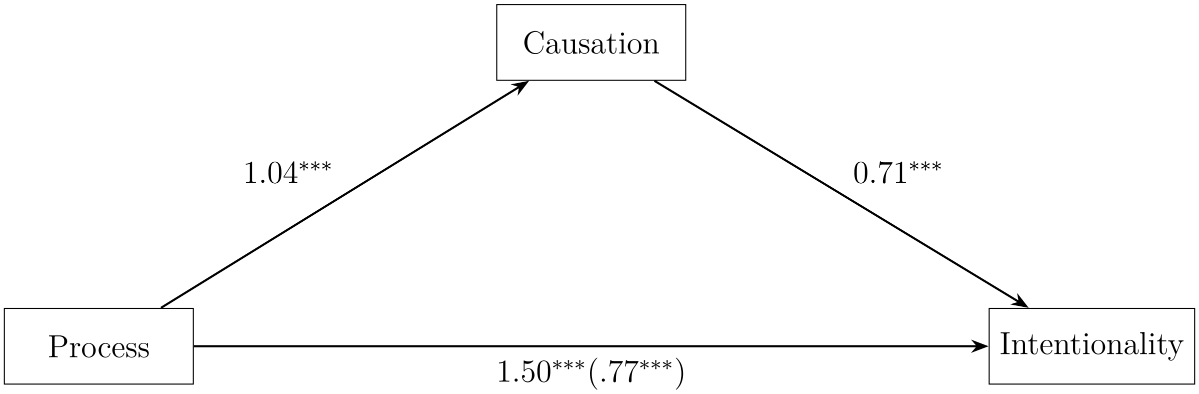

Figure S7

Mediation model for Study 3. Regression coefficients are computed from linear mixed models with participant-level random intercepts. The coefficient in parentheses represents the direct effect of Process on Intentionality.

References

Pavese, C., & Henne, P. (2023). The know-how solution to kraemer's puzzle. *Cognition*, *238*, 105490.

Rohrer, J. M., Hünermund, P., Arslan, R. C., & Elson, M. (2022). That's a lot to process! pitfalls of popular path models. *Advances in Methods and Practices in Psychological Science*, *5*(2), 25152459221095827.

Tingley, D., Yamamoto, T., Hirose, K., Keele, L., & Imai, K. (2014). Mediation: R package for causal mediation analysis. *Journal of statistical software*, *59*, 1–38.

Varghese, S., & Henne, P. (2025). Is there a simple explanation for the kraemer effect?
